# Long non-coding RNA NRSN2-AS1 promotes ovarian cancer progression through targeting PTK2/β-catenin pathway

**DOI:** 10.1038/s41419-023-06214-z

**Published:** 2023-10-24

**Authors:** Yi-Bo Wu, Shen-Yi Li, Jin-Yan Liu, Jia-Jia Xue, Jin-Fu Xu, Ting Chen, Tian-Yue Cao, Hui Zhou, Tian-Tian Wu, Chun-Lin Dong, Wei-Feng Qian, Long-Wei Qiao, Shun-Yu Hou, Ting Wang, Cong Shen

**Affiliations:** 1 Human Reproductive and Genetic Center, Affiliated Hospital of Jiangnan University, Wuxi, 214122 China; 2 Department of Obstetrics, Suzhou Municipal Hospital, The Affiliated Suzhou Hospital of Nanjing Medical University, Gusu School, Nanjing Medical University, Suzhou, 215002 China; 3 Department of Breast and Thyroid Surgery, Suzhou Municipal Hospital, The Affiliated Suzhou Hospital of Nanjing Medical University, Gusu School, Nanjing Medical University, Suzhou, 215002 China; 4 Suzhou Dushu Lake Hospital (Dushu Lake Hospital Affiliated to Soochow University), Suzhou, 215124 China; 5 State Key Laboratory of Reproductive Medicine, Department of Histology and Embryology, School of Basic Medical Sciences, Nanjing Medical University, Nanjing, 211166 China; 6 Department of Gynaecology, Suzhou Municipal Hospital, The Affiliated Suzhou Hospital of Nanjing Medical University, Gusu School, Nanjing Medical University, Suzhou, 215002 China; 7 State Key Laboratory of Reproductive Medicine, Center for Reproduction and Genetics, Suzhou Municipal Hospital, The Affiliated Suzhou Hospital of Nanjing Medical University, Gusu School, Nanjing Medical University, Suzhou, 215002 China

**Keywords:** Oncogenes, Ovarian cancer

## Abstract

As a common malignant tumor among women, ovarian cancer poses a serious threat to their health. This study demonstrates that long non-coding RNA NRSN2-AS1 is over-expressed in ovarian cancer tissues using patient sample and tissue microarrays. In addition, NRSN2-AS1 is shown to promote ovarian cancer cell proliferation and metastasis both in vitro and in vivo. Mechanistically, NRSN2-AS1 stabilizes protein tyrosine kinase 2 (PTK2) to activate the β-catenin pathway via repressing MG-53-mediated ubiquitinated degradation of PTK2, thereby facilitating ovarian cancer progression. Rescue experiments verify the function of the NRSN2-AS1/PTK2/β-catenin axis and the effects of MG53 on this axis in ovarian cancer cells. In conclusion, this study demonstrates the key role of the NRSN2-AS1/PTK2/β-catenin axis for the first time and explores its potential clinical applications in ovarian cancer.

## Introduction

Ovarian cancer (OC) is the most malignant gynecological cancer and seriously jeopardizes women’s health and quality of life [1, 2]. Owing to its nontypical symptoms in the early stages and its high invasiveness, OC has a 5-year patient survival rate of 20–40% [3, 4]. Identifying suitable biomarkers for diagnosing and treating OC is urgent right now.

Long-coding RNAs (lncRNAs), which are functional RNA molecules longer than 200 nucleotides without protein-coding ability, play a vital role in various cancers through multiple mechanisms [5, 6]. LncRNAs participate in gene regulation not only at the epigenetic and transcriptional levels [7] but also at the post-transcriptional and translational levels via interactions with RNAs [8–10] and proteins [11]. For example, p53-induced noncoding transcript (Pint) directly binds to polycomb repressive complex 2 (PRC2) and is required for H3K27 trimethylation during colon cancer progression [7]. You et al. found that PTAR in the cytoplasm acted as a competing endogenous RNA (ceRNA), interacting with miR-101 and regulating ZEB1 level, thereby promoting metastasis of OC [8]. Likewise, it has been proven that cytoplasmic SPOCD1-AS can induce OC metastasis through interacting with protein G3BP1 to remodel mesothelial cells [11].

The lncRNA NRSN2-AS1 (NCBI: NR_109990.1, Ensemble: ENSG00000225377) is situated on chromosome 20, spanning a total length of 5566-bp. Research on NRSN2-AS1 is limited. The latest research found that NRSN2-AS1 was located in both the cytoplasm and nucleus of cancer cells. Chen et al. have first showed that NRSN2-AS1 could facilitate ovarian cancer progression via sponging miR-744-5p to regulate PRKX expression [12]. Subsequently, Xu et al. found that NRSN2-AS1 promotes the progression of esophageal squamous cell carcinoma by regulating the ubiquitin degradation of PGK1 [13]. In a recent study conducted by Huang et al., the expression level of NRSN2-AS1 exhibited a significant positive correlation with immune cell infiltration and participated in the peroxisome and Peroxisome proliferator-activated receptor (PPAR) signaling pathways in hepatocellular carcinoma [14]. However, NRSN2-AS1 still needs to be further investigated in tumors for more in-depth functions.

In present study, we verified that NRSN2-AS1 was upregulated in OC tissues and cells, and positively corresponds with OC progression. Through RNA pull-down liquid chromatography-mass spectrometry (RNA pull-down LC-MS/MS), we found that NRSN2-AS1 could directly interact with PTK2 (protein tyrosine kinase 2). In depth, IP assays demonstrated that NRSN2-AS1 protected PTK2 from mitsugumin 53 (MG53)-mediated ubiquitinated degradation. Therefore, accumulated PTK2 enhances OC progression by activating the β-catenin pathway. The identification of the NRSN2-AS1/PTK2/β-catenin signaling pathway not only enriches our knowledge of the specific role of NRSN2-AS1 in OC but could also provide new targets for detecting and treating OC.

## Materials and Methods

### Bioinformatics analysis

The Cancer Genome Atlas (TCGA) datasets (http://cancergenome.nih.gov/) [15] were used to obtain clinical and RNA sequencing information on 419 OC and 88 normal ovarian tissue samples. The docking of NRSN2-AS1 bound to PTK2 were predicted with HDOCK (http://hdock.phys.hust.edu.cn/) and visualized with PyMOL (https://pymol.org/2/) as previously described [16, 17]. The cut-off employed to dichotomize OC patients in high and low NRSN2-AS1 expression is median.

### Samples collection

Fresh tumor and paracancerous tissues were derived from 23 OC patients with surgical resection from January 2021 to January 2022 at the Suzhou Municipal Hospital. All participants signed informed consent, and participant information will be fully protected. Approval was received from the Research Ethics Committee of Suzhou Municipal Hospital. Collected tissues were stored in RNA Keeper Tissue Stabilizer (Vazyme, Nanjing, China) at −80 °C as previously described [18].

### RNA extraction and real-time quantitative reverse transcription polymerase chain reaction (RT-qPCR) assays

Tissues fixed in RNA Keeper Tissue Stabilizer were ground to powder with liquid nitrogen. We extracted RNA with Total RNA Extraction Reagent (TRIzol, Vazyme) and reverse transcribed it into cDNA with HiScript III RT SuperMix for qPCR kit (Vazyme). The relative mRNA expression was measured using an AceQ qPCR SYBR Green Master Mix kit (Vazyme) on an Applied Biosystems 7500 RealTime PCR System. Finally, the expression level was analyzed with 2^–ΔΔCT^ and normalized to 18sRNA. Table S1 lists the primers used in this study.

### Fluorescence in situ hybridization (FISH) analysis

A tissue microarray with 48 OC and 10 normal tissue samples was purchased from Zhongke Huaguang Biotechnology Company (Shanxi, China). FISH assays were carried out with FISH kit (RiboBio Biotechnology, Guangzhou, China) following the manual. A unique probe targeting NRSN2-AS1 was synthesized by RiboBio. 4′,6-diamidino-2-phenylindole (DAPI, Beyotime, Haimen, China) was used for staining nuclei. All microscopy images were captured with a confocal laser microscope (LSM 810, Carl Zeiss, Oberkochen, Germany).

### Cell culture and treatments

Human OC cell lines (OVCAR3, A2780 and SKOV3) and normal ovarian epithelial cell lines (IOSE80) were obtained from the Chinese Academy of Cell Collection (Shanghai, China), and cultured in a humidified conditions at 37 °C with 5% CO_2_. IOSE80, SKOV3 and OVCAR3 cells were cultivated in RPMI-1640 (Gibco, USA) with fetal bovine serum (FBS) (ExCell Bio, New Zealand) and 1% penicillin/streptomycin (PS) (NCM Biotech, China). Specifically, 10%FBS was needed to IOSE80 and SKOV3, and 20% to OVCAR3. Dulbecco’s modified Eagle medium (Gibco, USA) with 10% FBS and 1% PS were used to culture A2780 cells.

When the cell fusion degree reached 60–70%, small interfering RNAs (siRNAs) (GenePharma, Suzhou, China) targeting NRSN2-AS1, PTK2, and MG53 as well as overexpression plasmids (GenePharma) pcDNA3.1-NRSN2-AS1, pEX-1-MG53 and an empty vector (EV) were transfected into OC cells by Lipofectamine 2000 (Invitrogen, USA) and X-treme GENE HP DNA transfection reagent (Mannheim, Germany). The nucleotide sequences of all siRNAs are shown on Table S2.

XAV-939 and SKL2001 (Selleck, Shanghai, China) were solubilized in dimethyl sulfoxide (DMSO) (Sigma Aldrich, USA) at 10 mM, and used in experiments at 15 μM. Following treatment, cells were harvested for further analysis 48 h later.

### Cell proliferation assays

A 96-well plate (2000 cells/well) was inoculated with the cells for the CCK-8 assay. Cell viability was measured every 24 h using a Cell Counting Kit-8 kit (Beyotime) on a microplate reader (Bio-Rad Model 680, USA) at 450 nm.

800–1000 cells were added into six-well plates for 2 weeks to evaluate cloning formation capabilities. The clones were fixed with methanol, stained with 0.1% crystal violet (Beyotime) and counted for analysis.

### Cell migration assays

In the transwell assay, 4.5 × 10^4^ cells in 300 μl serum-free medium were seeded into the upper chamber with 8 μm pore size (Corning, USA), whereas 700 μl complete medium was added into the lower chamber. After 48 h, the cells outside the chamber were fixed, stained and imaged for counting.

### Xenograft in mice

Four-week-old female athymic BALB/c nude mice (Vital River Laboratory, China) were used to construct a subcutaneous tumor model and lung metastasis model. Mice received 12 h light/12 h dark at 22–28 °C and 50–70% humidity under specific-pathogen-free conditions. The approval of mice investigations obtained from the Animal Ethics and Welfare Committee of Nanjing Medical University.

For the subcutaneous tumor model [19], 1 × 10^7^ cells transfected with sh-NRSN2-AS1 or sh-NC were subcutaneously injected into the left and right flanks of nude mice, respectively (*n* = 5). The tumor volumes were calculated every 3 days (V = 0.5 × D × d^2^ (V, volume; D, longitudinal diameter; d, latitudinal diameter)). We sacrificed the mice at 11 days, follow by removing and measuring the subcutaneous tumors.

For the lung metastasis model [20], the tail veins of mice were injected with 2 × 10^7^ treated cells (*n* = 5). An eight-week period ended with the mice being sacrificed, their lungs were removed and fixed in formalin.

### Immunofluorescence

Immunofluorescence assays were conducted as previous descriptions [21–24]. Briefly, the slides containing target cells and tissues were incubated with primary and Alexa-Fluor secondary antibodies (Thermo Scientific, Waltham, USA) orderly. Confocal laser microscopes (LSM 810) were used to observe all samples. A list of the primary antibodies used can be found in Table S3.

### Hematoxylin and eosin (H&E) staining

Staining of lung tissue sections with H&E was followed by ethanol dehydration, xylene hyalinization, and neutral balsam closure. A microscope (Axioskop 2 Plus, Zeiss) was used to photograph.

### RNA pull-down assays and LC-MS/MS analysis

T7 RNA polymerase (Ambio Life, Shanghai, China) and Biotin RNA Labeling Mix (Ambio Life) were used to transcribe and label NRSN2-AS1 according to the manual. Next, Pierce Magnetic RNA-Protein Pull-Down Kit (Thermo Scientific) was utilized to carry out RNA pull-down assays [25]. Finally, the proteins interacting with NRSN2-AS1 were identified by LC-MS/MS analysis.

### Western blot assays

We performed Western blotting according to our previous protocol [20, 26]. In short, radioimmunoprecipitation assay (RIPA, Beyotime) buffer containing 1% protease inhibitor phenylmethylsulfonyl fluoride (PMSF, Beyotime) was used to lysis protein samples. Subsequently, the proteins were denaturalized at 100 °C for 10 min, electrophoresed with 10% SDS-polyacrylamide gel (GenScript, China) and transferred to polyvinylidene difluoride membranes (Millipore, Billerica, USA). Primary antibodies and horseradish peroxidase-conjugated secondary antibodies were incubated on membranes. In the end, an Image-Pro Plus (Media Cybernetics, USA) was employed to measure the bands signal detected with the BeyoECL Plus kit (Beyotime). Tubulin antibody was used as a control. There is detailed information about the antibodies in Table S3. The original western blots were shown on the Original Data File of Supplemental Material.

### RNA immunoprecipitation (RIP) assays

RIP assays were conducted with Magna RIPTM RNA-Binding Protein immunoprecipitation kit (Millipore) as previously described [25]. Briefly, cell lysate, which lysed by RIP lysis buffer, was incubated with anti-PTK2 and anti-IgG antibodies at 4 °C. Subsequently, the protein–RNA complexes were obtained using 0.5 mg/ml proteinase K with 0.1% SDS. RT-qPCR were done to determine the interaction between PTK2 and NRSN2-AS1.

### TOP/FOP Flash luciferase assays

TOP/FOP Flash luciferase assays were performed to prove the effect of the NRSN2-AS1/PTK2 axis on β-catenin activity [12, 27, 28]. In brief, OVCANR3 and A2780 cells treated with si-NC, si-NRSN2-AS1, or si-PTK2 separately were co-transfected with TOP/Flash, FOP/Flash and Renilla luciferase plasmids (Beyotime) using Lipofectamine 2000. After 48 hours, the cells were lysed with the lysis buffer and the luciferase activities were detected with Luciferase Reporter Gene Assay Kit (Beyotime). The TOP/FOP-Flash values were normalized to Renilla luciferase activity.

### Protein half-life assays

Cycloheximide (CHX, 100 μg/ml) was added into OC cells to interrupt protein synthesis. Western blotting was conducted to detect the expression of PTK2 at 0, 1, 2, 4 h.

### Immunoprecipitation (IP) assays

IP assays were carried out according to our previously described procedure [16, 29]. Cell lysates were incubated with anti-PTK2 or anti-IgG antibodies overnight, followed by washed beads (Santa Cruz) for 2 h at 4 °C. Finally, the IP product, was detected by specific antibodies via western blotting. Antibody information is provided in Table S3.

### Ubiquitination assays

RIPA buffer containing 1% PMSF was used to lysed OC cells transfected with siRNAs or overexpression plasmids. Anti-PTK2 or anti-IgG antibodies were incubated overnight with cell lysates, followed by protein A/G beads for 2 h at 4 °C. Finally, the products were analyzed using immunoblotting with anti-Ub and anti-Ub-K48 antibodies.

### Statistical analysis

The data were provided as mean ± standard deviation and analyzed using Student’s t-tests for two groups, and one-way analyses of variance for three or more groups on SPSS 17.0 (SPSS, Inc., USA) and GraphPad Prism 7.0 (GraphPad Software, USA). Survival curves were profiled with Kaplan–Meier survival plots. *P* < 0.05 was represented as statistical significance.

## Results

### NRSN2-AS1 expression is positively correlated with OC progression

**Fig. 1:**
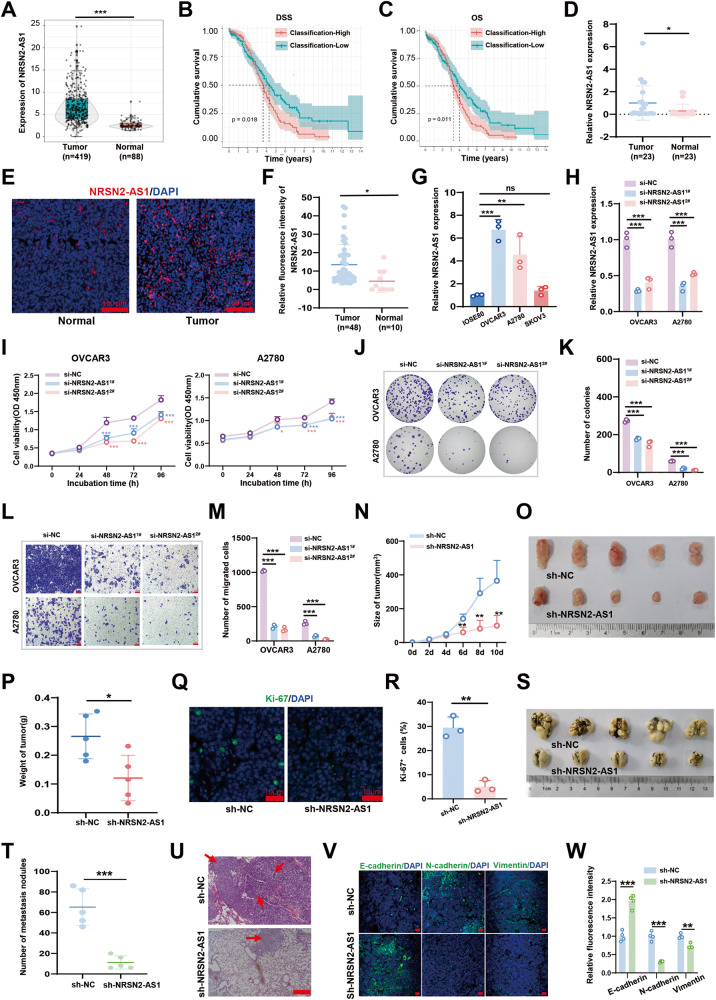
NRSN2-AS1 is significantly upregulated in OC and its levels are tightly correlated with OC progression both in vitro and in vivo. **A** TCGA data indicated differences in expression of NRSN2-AS1 between 419 tumor tissues and 88 normal tissues. **B, C** Kaplan–Meier curves showed a negative correlation between NRSN2-AS1 expression and disease-specific survival and overall survival in OC patients. **D** RT-qPCR was used to detect the relative expression of NRSN2-AS1 in 23 paired OC samples. **E** FISH assays showed the expression of NRSN2-AS1 (red) in OC tumor tissues (*n* = 48) and normal tissues (*n* = 10), with nuclei stained by DAPI (blue). Scale bar: 50 μm. **F** Quantification of fluorescence intensity from (**E**). **G** RT-qPCR was used to test NRSN2-AS1 expression in normal human ovarian epithelium cell (IOSE80) and OC cells (OVCAR3, A2780, and SKOV3), *n* = 3. **H** Relative expression of NRSN2-AS1 in OVCAR3 and A2780 cells transfected with si-NRSN2-AS1 and si-NC tested by RT-qPCR, *n* = 3. **I** The viability of OC cells was detected by CCK8 assays, *n* = 6. **J, K** The proliferation ability of NRSN2-AS1-transfected OC cells were determined with colony-formation assay, *n* = 3. **L, M** Transwell assays were done to investigate the migratory ability of OC cells, n = 3. Scale bar: 100 μm. **N-R** A total of 5 nude mice were injected with OVCAR3 cells transfected with sh-NC or sh-NRSN2-AS1. **N** Tumor volumes were calculated every 3 days. **O-P** Tumors were collected and weighted. **(Q, R)** Ki-67 expression in tumors were evaluated with immunofluorescence. Scale bar: 50 μm.**(S-W** OVCAR3 cells transfected with sh-NC or sh-NRSN2-AS1 were injected into the tails of nude mice (*n* = 5 each). **S** In both sh-NC and sh-NRSN2-AS1 mice, entire lungs were obtained, and **T** nodules on the lung surfaces were counted. **U** Lung sections were stained with H&E. **V** Relative expression of E-cadherin, N-cadherin, and vimentin in lung nodules was detected by immunofluorescence; *n* = 5. Scale bar: 50 μm. **W** Quantification of fluorescence intensity of (**V**). *P < 0.05, **P < 0.01, ***P < 0.001.

Based on TCGA data, NRSN2-AS1 was remarkably overregulated in OC tissues (Fig. 1A), and high NRSN2-AS1 level was associated with poor prognosis, including lower disease-specific survival and overall survival rates (Fig. 1B, C). Additionally, NRSN2-AS1, age and stage were found significantly associated with OC prognosis by multivariate Cox regression analysis (Fig. S1). High NRSN2-AS1 levels were also found in OC patient tissues, as verified by RT-qPCR and FISH assays (Fig. 1D–F). Relative to normal ovarian epithelial cells (IOSE80), the OVCAR3 and A2780 cell lines displayed higher levels of NRSN2-AS1 expression (Fig. 1G). Therefore, we used the OVCAR3 and A2780 cell lines for further analysis. Silencing NRSN2-AS1 using si-RNA strikingly decreased the cell viability, colony formation, and migration of both OVCAR3 and A2780 cells in vitro (Fig. 1H–M). Besides, smaller volumes and lower numbers of Ki67^+^ cells were observed in xenograft tumors in the sh-NRSN2-AS1 group. There are decreased metastatic nodules, increased E-cadherin positive cells, and reduced N-cadherin and vimentin-positive cells in the lung of the sh-NRSN2-AS1 group (Fig. 1N–W).

### NRSN2-AS1 interacts with PTK2

**Fig. 2:**
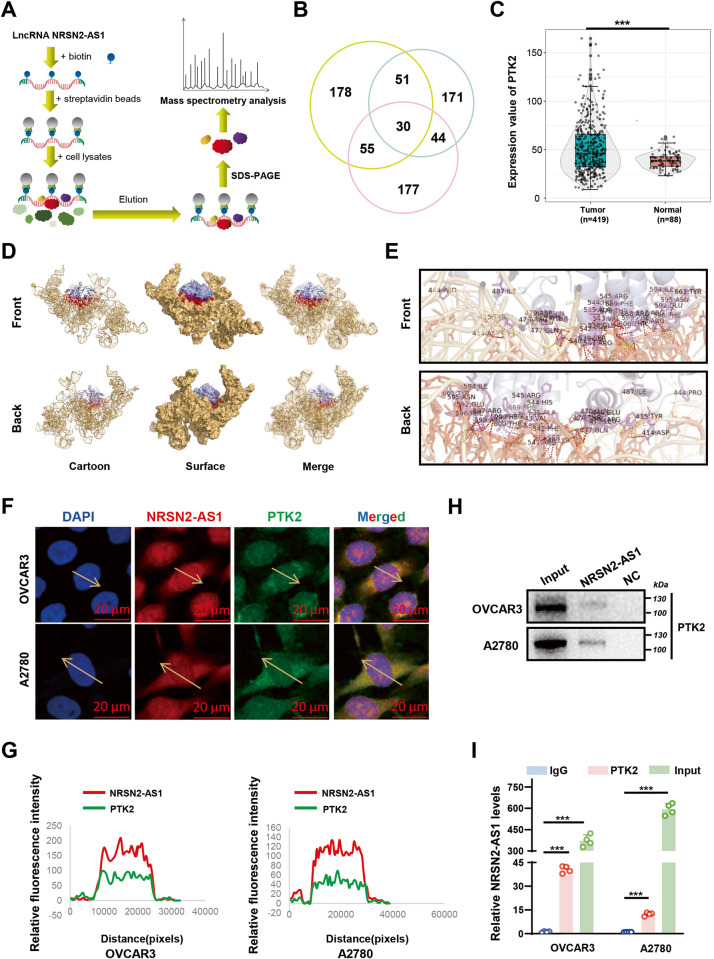
NRSN2-AS1 interacts with PTK2. **A** Flow chart of the RNA pull-down assays. **B** Venn diagram of proteins identified by MS from three independent RNA pull-down assays. **C** TCGA data indicated the expression of PTK2 in 419 tumor tissues and 88 normal tissues. **D** Visualization of the docking of NRSN2-AS1 (yellow) and PTK2 (blue) from front and back perspectives. Interacting residues are colored red for NRSN2-AS1 and purple for PTK2. The cartoon mode displays the backbone as well as the secondary structures of the corresponding proteins. The surface mode represents the solvent-accessible surface area. The merged mode combines cartoon and transparent surface views. **E** Visualization of the binding interface of the NRSN2-AS1/PTK2 complex. The interface view zooms and labels the residues (in stick mode) involved in the binding between NRSN2-AS1 and PTK2. **F, G** Co-immunostaining of NRSN2-AS1 and PTK2 in OC cells. Scale bar: 50 μm. **H** Western blot assays showed the expression levels of PTK2 after the NRSN2-AS1 pull-down assay. **I** RIP assays for NRSN2-AS1 binding to PTK2 in OC cells lysates, with rabbit anti-IgG as the negative control *(n* = 3). *P < 0.05, **P < 0.01, ***P < 0.001.

An RNA pull-down LC-MS/MS assay was performed to isolate potential proteins interacting with NRSN2-AS1 (Fig. 2A). From Venn diagrams of three independent RNA pull-down assays, 30 proteins overlapped (Fig. 2B and Table S4). These included PTK2, also known as focal adhesion kinase (FAK), which is reported to engaged in the progression of various tumor types. Based on TCGA data, we observed that PTK2 was expressed excessively in OC tissues than normal tissues (Fig. 2C).

Based on the three-dimensional (3D) structure, the interaction interface between NRSN2-AS1and PTK2 was predicted using HawkDock and visualized with PyMOL (Fig. 2D). The crossing surface was composed of residues 35–82 of NRSN2-AS1 and 414–670 of PTK2 (Fig. 2E and Table S5). Subsequently, the colocalization of NRSN2-AS1 and PTK2 in OC cells was confirmed by co-immunostaining (Fig. 2F, G). Furthermore, RNA-pulldown western blotting and RIP-qPCR experiments verified that NRSN2-AS1 interacted with PTK2 (Fig. 2H, I).

### PTK2 facilitates cell proliferation and migration and is associated with β-catenin signaling activation in OC cells

**Fig. 3:**
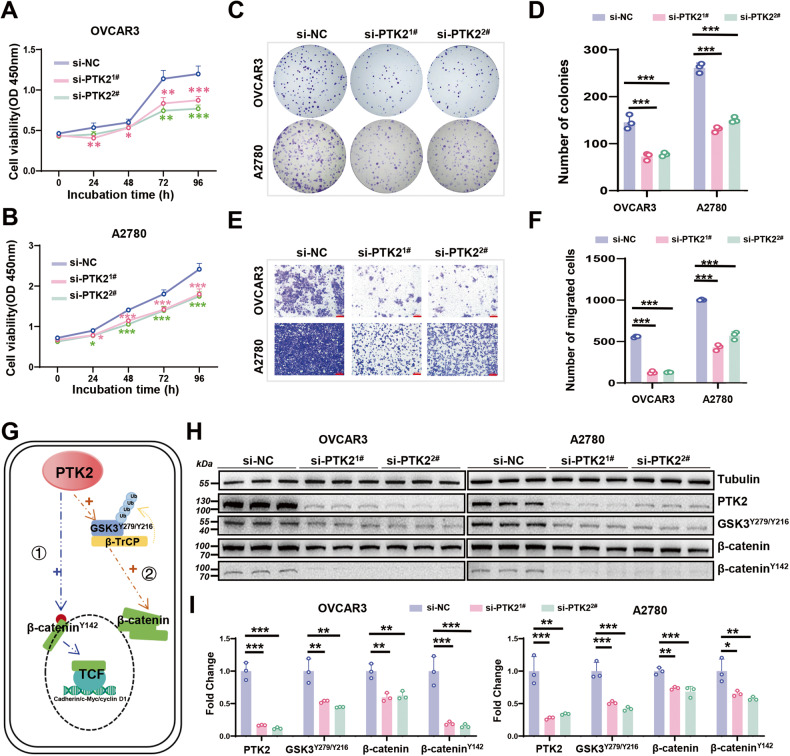
Effects and underlying mechanisms of PTK2 in OC cells. **A, B** CCK8 assays detected the OC cells viability with PTK2 silencing, *n* = 6. **C, D** The proliferation ability of cells transfected with si-PTK2 was assessed by colony formation assays, *n* = 3. **E, F** The migration abilities of cells transfected with si-PTK2 were examined by transwell assays, *n* = 3. Scale bar: 100 μm. **G** Schematic diagram of the pathway from PTK2 to β-catenin. **H, I** Western blots to determine the expression of PTK2, GSK3^Y279/Y216^, β-catenin, and β-catenin^Y142^ in PTK2-downregulated OC cells, *n* = 3. *P < 0.05, **P < 0.01, ***P < 0.001.

OVCAR3 and A2780 cells were transfected with si-PTK2 or si-NC, respectively to explore the effect of PTK2 on OC cells. The downregulation of PTK2 reduced cell proliferation, including cell viability and colony formation (Fig. 3A–D). The migratory capacity was also dramatically reduced after PTK2 silencing (Fig. 3E, F). In short, the knockdown of PTK2 suppressed OC cell proliferation and migration in vitro.

β-catenin signaling is already being implicated in multiple aspects of human cancers, including tumorigenesis, progression, and malignant invasion [30]. In various cancers [31–33], β-catenin is abnormally activated, including OC [34, 35]. Most β-catenin is located in the cytoplasm, where it maintains cell-cell adhesion through interactions with E-cadherin [36]. In the nucleus, β-catenin binds T-cell factor/lymphoid enhancer to activate downstream genes, causing uncontrolled proliferation and migration of neoplastic cells [37]. As illustrated (Fig. 3G), studies have demonstrated that PTK2 participates in activating β-catenin signaling. On the one hand, PTK2 can directly phosphorylate β-catenin^Y142^ to promote its translocation into the nucleus [38, 39]. On the other hand, it phosphorylates GSK3^Y279/Y216^ to promote its ubiquitinated degradation mediated by E3 ligase β-TrCP (β-transducin repeats-containing proteins), thereby stabilizing the expression of total β-catenin [40] (Fig. 3G). Through western blotting, we found that PTK2 silencing resulted in decreased expression of β-catenin^Y142^, GSK3^Y279/Y216^, and total-β-catenin in OVCAR3 and A2780 cells (Fig. 3H, I). Overall, the above results indicate that PTK2 might also trigger the β-catenin pathway in OC cells.

### Knockdown of NRSN2-AS1 reduces protein expression of PTK2 and blocks β-catenin activation

**Fig. 4:**
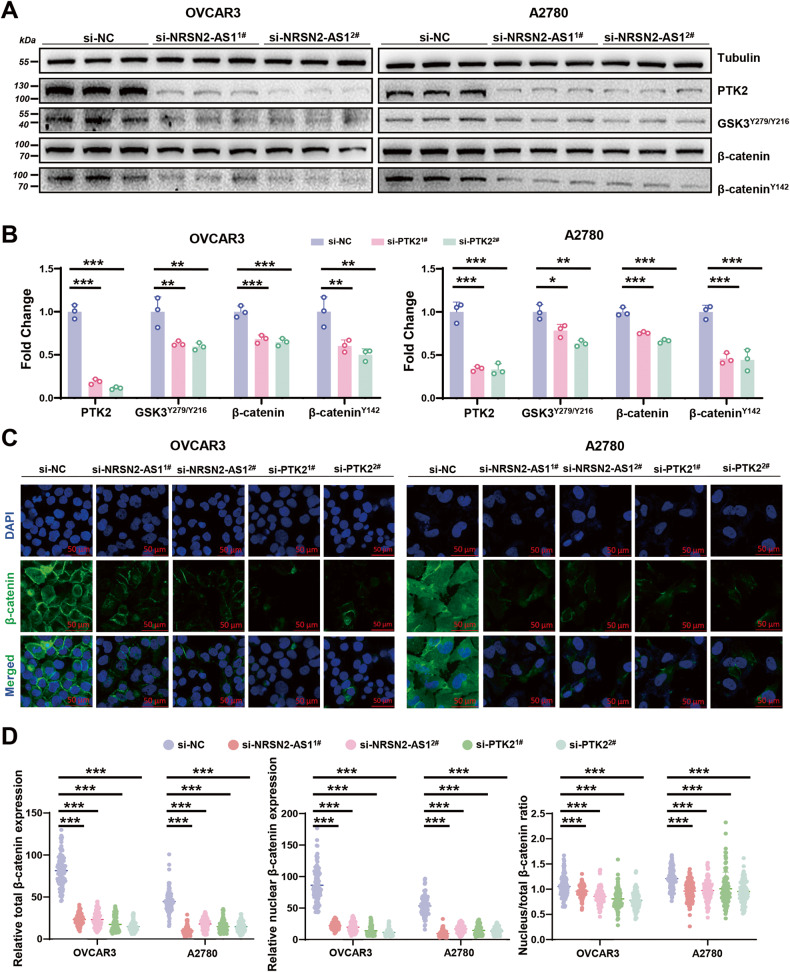
Potential molecular mechanisms involving NRSN2-AS1 and PTK2/β-catenin pathway in OC cells. **A, B** An internal control was tubulin, western blotting was used to determine the protein level of PTK2, GSK3^Y279/Y216^, β-catenin, and β-catenin^Y142^ in OC cells with silencing of NRSN2-AS1, *n* = 3. **C** Immunofluorescence experiments were performed to detect β-catenin expression levels (green) in the si-NRSN2-AS1 and si-PTK2 groups; DAPI was used to stain nuclei. Scale bar: 50 μm. **D** Quantification of fluorescence intensity from (**C**), *n* = 100 cells for each group. *P < 0.05, **P < 0.01, ***P < 0.001.

Western blot was applied to clarify the influences of NRSN2-AS1 on the PTK2/β-catenin pathway. Reduced PTK2, β-catenin^Y142^, GSK3^Y279/Y216^, and total β-catenin expression were observed in OC cells with NRSN2-AS1 silencing (Fig. 4A, B). Furthermore, immunofluorescence confirmed that silencing either NRSN2-AS1 or PTK2 resulted in a dramatic loss of β-catenin expression. The reduction was more pronounced in the nucleus (Fig. 4C, D). Aside from that, the knockdown of NRSN2-AS1 or PTK2 produced a remarkable reduction of TOP/FOP luciferase activity (Fig. S2). Therefore, the knockdown of both NRSN2-AS1 and PTK2 blocks β-catenin activation.

### NRSN2-AS1 promotes OC proliferation and migration in a manner dependent on β-catenin signaling

**Fig. 5:**
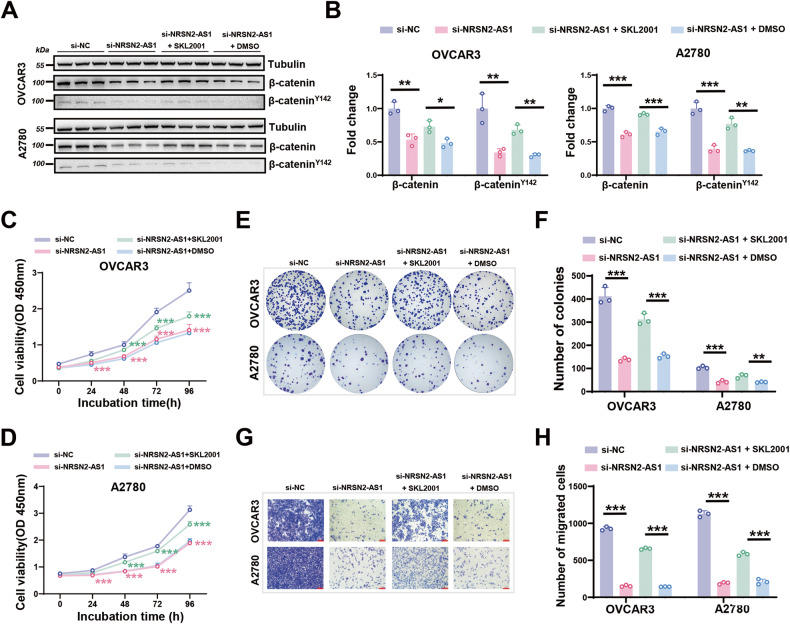
Effects of NRSN2-AS1/β-catenin pathway in OC cells. OC cells were transfected with si-NC, si-NRSN2-AS1, si-NRSN2-AS1 + SKL2001 (β-catenin activator, 15 μM), or si-NRSN2-AS1 + DMSO. **A, B** Western blot was used to identify the expression of β-catenin and β-catenin^Y142^, *n* = 3. **C, D** CCK8 assays to detect cell viability, *n* = 6. **E, F** The cells proliferation ability was determined by colony formation assays, *n* = 3. **G, H** Investigating migratory abilities with transwell assays, *n* = 3. Scale bar: 100 μm. *P < 0.05, **P < 0.01, ***P < 0.001.

To further verify the key role of β-catenin in OC progression associated with NRSN2-AS1, SKL2001 [41], a small-molecule β-catenin agonist, was applied to OVCAR3 and A2780 cells with silencing NRSN2-AS1. The expression of both β-catenin^Y142^ and total β-catenin was significantly enhanced after treatment with SKL2001 (Fig. 5A, B). In addition, loss-of-function assays revealed that activation of β-catenin in the NRSN2-AS1 knockdown group could reverse cell proliferation and migration in vitro (Fig. 5C–H).

### NRSN2-AS1 promotes OC progression through targeting PTK2/β-catenin axis

**Fig. 6:**
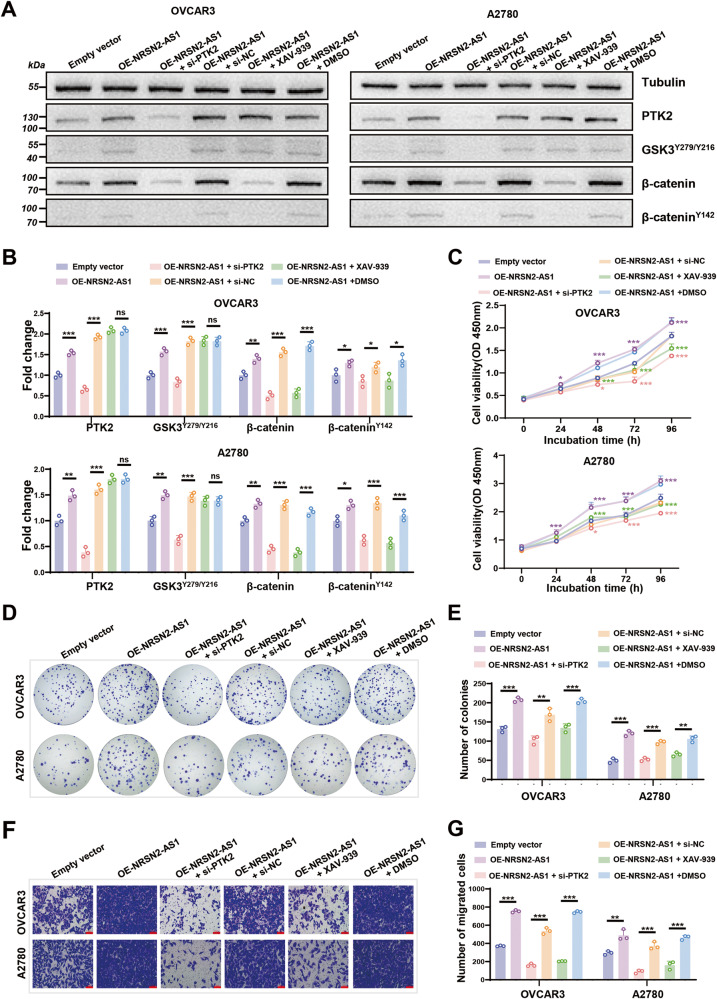
Effects of NRSN2-AS1/PTK2/β-catenin pathway in OC cells. OC cells were transfected with EV, OE-NESN2-AS1, OE-NRSN2-AS1+si-PTK2, OE-NRSN2-AS1+si-NC, OE-NRSN2-AS1 + XAV-939 (β-catenin inhibitor, 15 μM), or OE-NRSN2-AS1 + DMSO. **A, B** Western blot analysis of the expression of PTK2, GSK3^Y279/Y216^, β-catenin and β-catenin^Y142^, *n* = 3 for each group. **C** CCK8 assays were performed to assess cell viability, *n* = 6. **D, E** Colony formation assays were done to detect proliferation ability, *n* = 3. **F, G** Transwell assays were used to investigate changes in migratory abilities, *n* = 3. Scale bar: 100 μm. *P < 0.05, **P < 0.01, ***P < 0.001.

To further prove the engagement of the PTK2/β-catenin axis in NRSN2-AS1-mediated OC progression, si-PTK2 and XAV-939 [42], a small-molecule β-catenin inhibitor, were applied to OVCAR3 and A2780 cells overexpressing NRSN2-AS1. As a result, the expression of PTK2, GSK3^Y279/Y216^, β-catenin^Y142^, and total β-catenin were significantly increased after overexpression of NRSN2-AS1, whereas silencing of PTK2 reduced the expression of PTK2, GSK3^Y279/Y216^, β-catenin^Y142^, and total β-catenin. Moreover, depression of β-catenin via XAV-939 also diminished the protein level of β-catenin^Y142^ and total β-catenin but not PTK2 or GSK3^Y279/Y216^ (Fig. 6A, B). Furthermore, cell function assays demonstrated that inhibition of both PTK2 and β-catenin noticeably abolished the proliferation and migration of OC cells overexpressing NRSN2-AS1 (Fig. 6C–G).

According to OC patients cohort downloaded from the TCGA database, there were significant positive correlation between NRSN2-AS1 and PTK2, PTK2 and β-catenin, and NRSN2-AS1 and β-catenin (Fig. S3A). Moreover, K-M survival analyses with the integrated NRSN2-AS1/PTK2/β-catenin gene signature demonstrated that the higher the expression, the worse the overall survival in OC (Fig. S3B). Additionally, time-dependent receiver operating characteristic (ROC) curve analyses further elucidated the potential prognostic value of the integrated NRSN2-AS1/PTK2/β-catenin gene signature. At 3, 5 and 8 years the Area Under Curves (AUCs) were 0.520, 0.580 and 0.683 (Fig. S3C).

Metastatic progression heavily relies on the EMT procedure. To investigate the involvement of the NRSN2-AS1/PTK2/β-catenin network in modulating the expression of typical mesenchymal/epithelial markers in OC cells, the RT-qPCR analyses of the NRSN2-AS1/PTK2/β-catenin network on EMT markers were carried out. As a result, silencing NRSN2-AS1 could downregulate the expression of some mesenchymal markers (TWIST, NCAD, SNAIL, SLUG, ZEB1) and upregulate epithelial markers (ECAD), and SKL2001 could reverse it partially in OVCAR3 and A2780 (Fig. S2A) (Fig. S4A). Contrarily, overexpressed NRSN2-AS1 could promote EMT, whereas si-PTK2 and XAV939 could partially reverse it (Fig. S4B). A trend to reduction/increase on EMT were detected with the change of NRSN2-AS1/PTK2/β-catenin network, although some differences were not statistically significant.

Finally, we analyzed the expression of PTK2 and β-catenin in xenograft tumors derived from OVCAR3 cells. As showed by immunofluorescence, there were reduced PTK2 and β-catenin signals in the sh-NRSN2-AS1 group, when compared with these in control group (Fig. S4C, D).

Based on these results, it appears that NRSN2-AS1 promotes OC progression by targeting the PTK2/β-catenin axis.

### NRSN2-AS1 protects PTK2 from polyubiquitinated degradation by E3 ligase MG-53 in OC

**Fig. 7:**
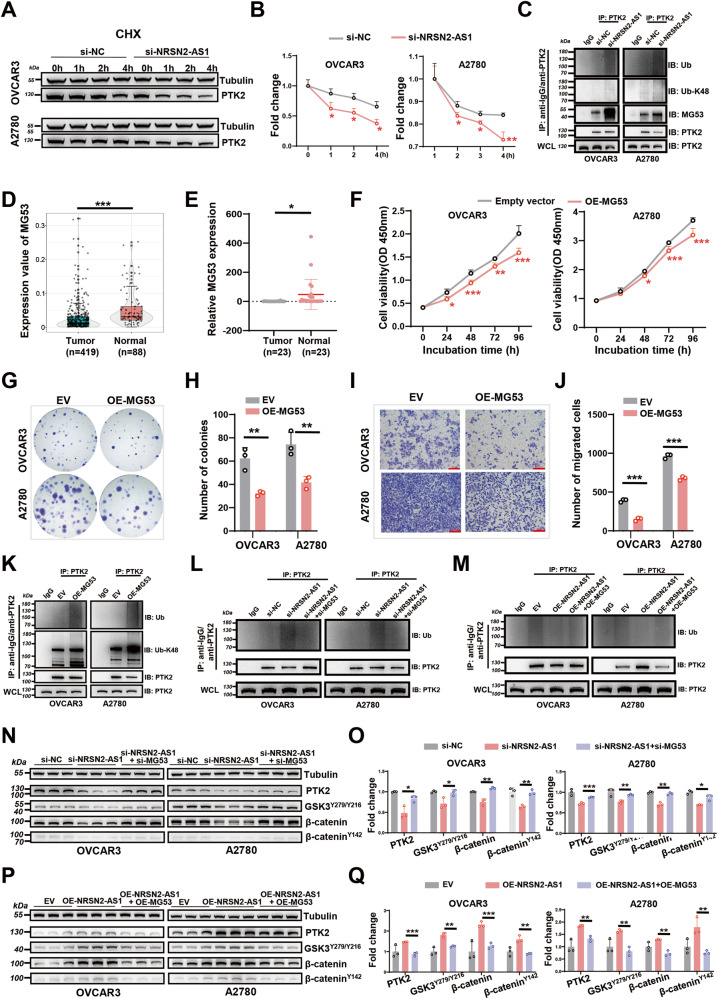
Effects of MG53 on NRSN2-AS1/PTK2/β-catenin pathway in OC cells. **A** OC cells treated with CHX for 0 h, 1 h, 2 h, and 4 h were analyzed by western blotting for PTK2 expression. **B** Quantification of results from (**A**), *n* = 3. **C** After MG132 (20 μM) treatment for 6 h, IP assays were performed with anti-IgG or anti-PTK2 in OVCAR3 and A2780 cells transfected with si-NRSN2-AS1. Immunoblot (IB) analysis of total-ubiquitin (Ub), Lys-48 ubiquitin (Ub-K48), MG53 and PTK2 in IgG- and PTK2-immunoprecipitated products and whole-cell lysate (WCL). **D** Expression of MG53 in 419 tumor and 88 normal tissues based on TCGA. **E** Relative mRNA level of MG53 was detected in 23 paired OC tissues by RT-qPCR. **F** CCK8 assays were used to verified OC cell viability after overexpressing MG53 (*n* = 6). **G, H** Colony formation assays were used to determine the proliferation ability (n = 3). **I, J** Transwell assays were used to investigate OC cell migration (*n* = 3). Scale bar: 100 μm. **K** IB analysis of Ub and Ub-K48 levels of PTK2 in IgG- and PTK2-immunoprecipitated products and WCL-derived cells transfected with EV and OE-MG53. **L** IB analysis of Ub level of PTK2 in IgG- and PTK2-immunoprecipitated products and WCL-derived cells transfected with si-NC, si-NRSN2-AS1, or si-NRSN2-AS1+si-MG53. **M** IB analysis of Ub level of PTK2 in IgG- and PTK2-immunoprecipitated products and WCL-derived cells transfected with EV, OE-NRSN2-AS1, or OE-NRSN2-AS1 + OE-MG53. **N, O** With tubulin as an internal control, western blot analysis was used to determine the expression of PTK2, GSK3^Y279/Y216^, β-catenin, and β-catenin^Y142^ in OC cells transfected with si-NC, si-NRSN2-AS1, or si-NRSN2-AS1+si-MG53, *n* = 3 for each group. **P, Q** The protein level of PTK2, GSK3^Y279/Y216^, β-catenin, and β-catenin^Y142^ were determined by western blot in OC cells transfected with EV, OE-NRSN2-AS1, or OE-NRSN2-AS1 + OE-MG53, *n* = 3 for each group. *P < 0.05, **P < 0.01, ***P < 0.001.

In this study, we observed that NRSN2-AS1 affected the protein level of PTK2 (Fig. 4A, B). We therefore assessed the stability of PTK2 in the absence of NRSN2-AS1 via CHX assays in OC cells. The protein stability of PTK2 significantly diminished after downregulation of NRSN2-AS1 (Fig. 7A, B). Furthermore, IP assays showed that OC cells transfected with si-NRSN2-AS1 displayed higher levels of polyubiquitination of PTK2. (Fig. 7C). The proteasome could specifically recognize substrate proteins labeled with the K48 ubiquitin chain, which in turn degrades them. In this study, it was obvious that Lys-48 was the critical lysine for PTK2 ubiquitination (Fig. 7C). The striated muscle-specific MG53, also known as tripartite motif-containing 72 (TRIM72), has been reported to induce PTK2 ubiquitination and degradation during skeletal myogenesis [43]. In our study, IP assays also verified that the interaction between MG53 and PTK2 was enhanced in NRSN2-AS1-knockdown OC cells (Fig. 7C), which raised the possibility that NRSN2-AS1 might stabilize PTK2 through blocking the ubiquitination and degradation mediated by MG53 in OC cells.

Based on TCGA data (Fig. 7D) and 23 paired clinical samples (Fig. 7E), OC tissues had significantly lower MG53 levels than normal tissues. Furthermore, gain-of-function assays demonstrated that MG53 inhibited OC cell proliferation and migration in vitro (Fig. 7F–J). These results suggest that MG53 appears to play a tumor-suppressive role in OC based on all of these results.

To clarify the function of MG53 in the interaction between NRSN2-AS1 and PTK2, IP assays were carried out. We found that MG53 markedly promoted both total and K48-linked polyubiquitination of PTK2 in OC cells (Fig. 7K). Furthermore, the knockdown of MG53 reduced the increase in ubiquitination level of PTK2 caused by NRSN2-AS1 silencing in OC cells (Fig. 7L). Similarly, NRSN2-AS1 lost the ability to inhibit PTK2 ubiquitination when MG53 was simultaneously overexpressed (Fig. 7M).

Finally, western blot assays showed that silencing MG53 could recover the expression of PTK2/β-catenin induced by NRSN2-AS1 silencing (Fig. 7N, O), whereas overexpression of MG53 diminished the PTK2/β-catenin signaling activated by NRSN2-AS1 upregulation (Fig. 7P, Q).

As a consequence, we conclude that NRSN2-AS1 regulates the PTK2/β-catenin pathway in a manner that inhibits PTK2 polyubiquitination and degradation induced by MG-53 in OC cells.

## Discussion

Recent evidence suggests that the novel oncogene NRSN2-AS1 is hyperexpressed in esophageal squamous cell carcinoma [13] and OC tissues [12], and promotes OC cells proliferation and migration via interaction with miR-744-5p to modulate PRKX. In this study, we further demonstrated that NRSN2-AS1 is highly expressed in OC tissues based on analysis of patient samples and OC tissue microarrays. Through RNA pull-down LC-MS/MS assays, we found that NRSN2-AS1 could interact with many proteins in addition to its previously reported role as a ceRNA. Among this, PTK2 was first identified as a tyrosine-phosphorylated protein located at focal adhesions [44, 45]. Over the decades, evidence suggests that PTK2 is implicated in cell adhesion, migration, invasion, survival, and proliferation via kinases or without them [46, 47]. Most studies have found that PTK2 is often upregulated in solid epithelial cancers, where it contributes to tumor malignant behavior [40, 48–50]. As a susceptibility gene for OC [51], PTK2 is frequently overexpressed in OC and is associated with immunosuppression [52] and chemotherapy resistance [53, 54]. Downregulation of PTK2 inhibits OC growth in vivo [55, 56]. Thus, in this study, PTK2 was selected as a candidate protein regarding its interactions with NRSN2-AS1 in OC. Previously, studies have shown that lncRNAs could influence the activation of the PTK2 pathway, thereby facilitating tumor advancement [57–62]. For example, lncRNA KCNQ1OT1 regulates proliferation and cisplatin resistance in tongue cancer via miR-211-5p mediated Ezrin/Fak/Src signaling [63]. Nevertheless, there was no evidence for a direct binding between lncRNAs and PTK2 protein. In this study, we propose this interaction for the first time. 3D structures, co-localization analysis, RNA-pulldown western blotting, and RIP-qPCR experiments were further verified the direct interaction between NRSN2-AS1 and PTK2. Furthermore, the proliferation and migration of OC cells were suppressed by PTK2 silencing. A key role of NRSN2-AS1/PTK2 axis in OC cells seems to be revealed by these results.

Numerous investigations have demonstrated that the β-catenin pathway governs many aspects of OC development, including stem cell self-renewal, metastasis, chemoresistance, angiogenesis, and immune evasion [64]. It has reported that PTK2 could participate in the β-catenin pathway in two ways. First, activated PTK2 could localize at cell junctions and phosphorylate β-catenin^Y142^ directly [38, 39]. Second, PTK2 phosphorylates GSK3^Y279/Y216^ to stabilize β-catenin, thereby promoting tumorigenesis [40]. Nevertheless, the relevance of PTK2 and β-catenin in OC has remained obscure. In this study, we found that PTK2 activated β-catenin both directly and indirectly in OC cells. More importantly, NRSN2-AS1 exerted a pro-cancer effect in OC cells through stabilizing PTK2 and subsequently promoted PTK2/β-catenin pathway activation.

We then considered how NRSN2-AS1 regulated the stability of PTK2. Previous studies have reported that PTK2 could been degraded in a polyubiquitylation-related manner via the E3 ligase MG53 during myogenesis [43, 65]. Our results also indicated that silencing of NRSN2-AS1 could enhance the degradation of PTK2 depending on MG53-mediated ubiquitination in OC cells. Several reports have shown that MG53 inhibits tumor progression in non-small-cell lung [66], tongue [67], colorectal [68], and hepatocellular [69] cancers. Here, we clarified the tumor suppressor role of MG53 in OC. Furthermore, rescue experiments showed that NRSN2-AS1 regulates the PTK2/β-catenin pathway in a manner that blocks ubiquitinated degradation of PTK2 mediated by MG-53 in OC.

There has been less research on the potential applications of PTK2 and theβ-catenin pathway in cancer therapies. A few preclinical and clinical trials (I, Ib, or II) have shown that PTK2 inhibitors could enhance activity in combination with cytotoxic drugs or agents targeting angiogenesis [65, 70–72]. Zhai and colleagues reported that APG-2449, a novel ALK/ROS1/FAK inhibitor, is effective against human ovarian tumor either alone or in combination with other therapies [73]. Moreover, researchers have studied many potential compounds targeting the β-catenin signaling pathway in pre-clinical and phase I/II trials [74]. For instance, WNT974 has been shown to produce cytostatic effects in ascites cells from patients with primary OC [75]. Despite various pre-clinical evaluations of potential therapies targeting PTK2 and β-catenin, clinical applications still face significant challenges.

**Fig. 8:**
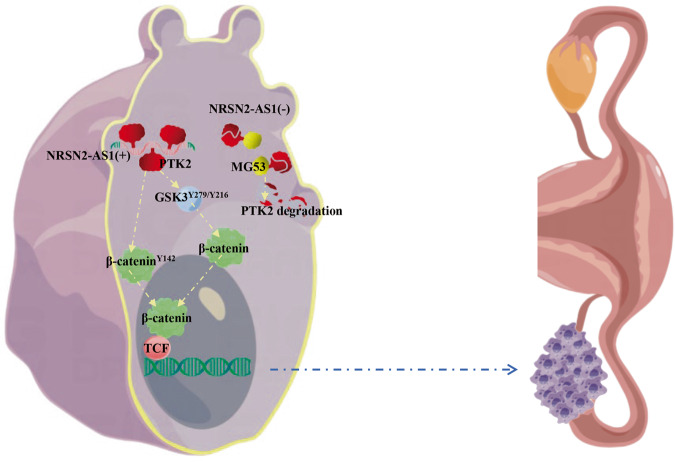
Schematic diagram of the mechanism by which NRSN2-AS1 promotes OC progression. NRSN2-AS1 interacts with PTK2 and protects it from MG53-mediated degradation, thereby activating the β-catenin pathway and promoting ovarian cancer progression.

This study describes a novel mechanism by which the NRSN2-AS1/PTK2/β-catenin axis underlies OC progression (Fig. 8). NRSN2-AS1 interacts with PTK2 and protects it from MG53-mediated polyubiquitination and subsequent degradation, thereby regulating the activity of β-catenin. In the absence of NRSN2-AS1, MG53 mediates the degradation of PTK2 via ubiquitination signaling. Despite its significant results, this study had some shortcomings. All clinical specimens came from the same source and were relatively few. Therefore, the findings regarding clinical features such as TNM or grade cannot be generalized. Multi-center studies with a larger sample size are needed. However, this study provides a better understanding to the role of NRSN2-AS1 in the PTK/β-catenin pathway in OC. Further study of the clinical implications of the NRSN2-AS1/PTK2/β-catenin pathway may contribute to early OC diagnosis and treatment.

### Supplementary information

Supplemental material

Origial Data File

checklist

## Data Availability

All data are available from the corresponding author upon reasonable request.
