# Supplementary material for: Long non-coding RNA NRSN2-AS1 promotes ovarian cancer progression through targeting PTK2/β-catenin pathway
Source: Cell Death Dis. 2023 Oct 24;14(10):696. doi: 10.1038/s41419-023-06214-z (PMC10598275; doi:10.1038/s41419-023-06214-z)
Supplement: Supplementary file 1 — Supplemental material [file 41419_2023_6214_MOESM1_ESM.pdf]

SUPPLEMENTARY MATERIAL

Table S1: The primers sequence used in the study.

Table S2: The sequence of siRNAs used in this study.

Table S3: The information of antibodies used in this study.

Table S4: Putative inter-actors identified from three independent RNA pull-down assays in this study.

Table S5: The potential structural sites between NRSN2-AS1 and PTK2.

Figure S1: The results of multivariate Cox regression analysis.

Figure S2: NRSN2-AS1/PTK2 regulates β-catenin signaling pathway in ovarian cancer.

Figure S3: NRSN2-AS1/PTK2/β-catenin axis was associated with poor prognosis of OC patients.

Figure S4: The effect of NRSN2-AS1/PTK2/β-catenin axis on ovarian cancer cells.

Table S1 The primers sequence used in the study.

Primers	Sequences
18sRNA-F	5'-AAACGGCTACCACATCCAAG-3'
18sRNA-R	5'-CCTCCAATGGATCCTCGTTA-3'
NRSN2-AS1-F	5'-CCTGCTTCCATCTGACGCTG-3'
NRSN2-AS1-R	5'-ATGTCCTCTCTTCCAGGGAGTT-3'
MG53-F	5'-TGTGGTCTCCTGTGTTGCTC-3'
MG53-R	5'-GACCCCAGCAGTTCAGTTCA-3'

Table S2 The sequence of siRNAs used in this study.

si-RNAs	Sequences
si-NC	5'-UUCUCCGAACGUGUCAGGU-3'
si-NRSN2-AS1[1#]	5'-CGAGCCUGCUUCCAUCUGA-3'
si-NRSN2-AS1[2#]	5'-GGCCGAGACACUCACUCUU-3'
si-PTK2 [1#]	5'-CCUAAGAGUUUACUGGAUU-3'
si-PTK2 [2#]	5'-CCUGUAUGCCUAUCAGCUU-3'
si-MG53	5'-CCAGAAUACUGACAAGCGU-3'

Table S3 The information of antibodies used in this study.

Antigen	Source	Company
Tubulin	Mouse	Beyotime
PTK2	Rabbit	Cell Signaling Technology
GSK3 (Tyr279/Tyr216)	Mouse	Miliipore
β-catenin	Rabbit	Cell Signaling Technology
β-catenin (Y142)	Rabbit	Abcam
MG53	Rabbit	Proteintech
Ub	Rabbit	Santa Cruz Blotechnology
Ub-K48	Rabbit	Abcam
Ki-67	Rabbit	Abcam
N-cadherin	Rabbit	Proteintech
E-cadherin	Rabbit	Proteintech
Vimentin	Rabbit	Cell Signaling Technology

Table S4 Putative inter-actors identified from three independent RNA pull-down assays in this study.

Protein IDs	Protein names	Gene names	iBAQ Ctr-1	iBAQ Ctr-2	iBAQ Ctr-3	iBAQ NRSN2-AS1-1	iBAQ NRSN2-AS1-2	iBAQ NRSN2-AS1-3
A0A087WTD7;A8MYJ1;Q12802-4;Q12802; Q12802-2;A0A087WY36	A-kinase anchor protein 13	AKAP13	0	0	0	287860	316840	230430
M0R0I0;J3KQS6;Q9NWV8-3;Q9NWV8; M0QXG9;M0R3F4;M0R2A4	BRISC; BRCA1-A complex member 1	BABAM1	0	0	0	496330	964080	497650
H0YNU5;A0A3B3IT82;P54132	Bloom syndrome protei n	BLM	0	0	0	667660	869710	844840
P36551;H0YA22	Oxygen-dependent coproporphyrinogen- III oxidase, mitochondrial	CPOX	0	0	0	346670	1586100	1879500
Q92620;Q92620-2	Pre-mRNA-splicing factor ATP-dependent RNA helicase PRP16	DHX38	0	0	0	272980	153570	104680
Q9H2P9-3;Q9H2P9-2;Q9H2P9-6;Q9H2P9-4; Q9H2P9;Q9H2P9-5	Diphthine synthase	DPH5	0	0	0	1479600	2446800	1967500
Q96FJ2;P63167	Dynein light chain 2, cytoplasmic; Dynein light chain 1, cytoplasmic	DYNLL2; DYNLL1	0	0	0	1528100	2699300	1830700
P81605;P81605-2	Dermcidin;Survival-promoting peptide; DCD-1	DCD	0	0	0	23076000	8011400	24335000
Q9H223	EH domain-containing protein 4	EHD4	0	0	0	1085800	797240	648760
O75955;O75955-2;A0A140T9R1;A2AB10; A2AB12;A0A0G2JJQ6	Flotillin-1	FLOT1	0	0	0	304230	1153500	931110

Table S5 The potential structural sites between NRSN2-AS1 and PTK2.

Receptor name	Receptor position	Receptor element	Ligand name	Ligand position	Ligand element	Distance (Å)
PTK2	542	PHE	NRSN2-AS1	77	G	2.007
PTK2	598	ARG	NRSN2-AS1	76	U	2.132
PTK2	670	THR	NRSN2-AS1	82	U	2.351
PTK2	596	PHE	NRSN2-AS1	73	U	2.652
PTK2	476	ARG	NRSN2-AS1	36	A	2.69
PTK2	596	PHE	NRSN2-AS1	76	U	2.722
PTK2	543	VAL	NRSN2-AS1	78	U	2.726
PTK2	539	SER	NRSN2-AS1	82	U	2.855
PTK2	477	GLN	NRSN2-AS1	77	G	2.959
PTK2	598	ARG	NRSN2-AS1	73	U	2.973
PTK2	595	ASN	NRSN2-AS1	38	A	3.1
PTK2	542	PHE	NRSN2-AS1	78	U	3.13
PTK2	541	ARG	NRSN2-AS1	82	U	3.179
PTK2	661	TYR	NRSN2-AS1	72	U	3.324
PTK2	474	THR	NRSN2-AS1	77	G	3.341
PTK2	541	ARG	NRSN2-AS1	78	U	3.388
PTK2	596	PHE	NRSN2-AS1	38	A	3.452
PTK2	597	ARG	NRSN2-AS1	76	U	3.488
PTK2	596	PHE	NRSN2-AS1	75	A	3.627
PTK2	545	ARG	NRSN2-AS1	78	U	3.821
PTK2	540	LYS	NRSN2-AS1	77	G	3.869
PTK2	600	THR	NRSN2-AS1	78	U	3.916
PTK2	414	ASP	NRSN2-AS1	36	A	3.922
PTK2	668	ARG	NRSN2-AS1	82	U	4.01
PTK2	669	PHE	NRSN2-AS1	82	U	4.027
PTK2	478	PHE	NRSN2-AS1	77	G	4.092
PTK2	545	ARG	NRSN2-AS1	76	U	4.236
PTK2	541	ARG	NRSN2-AS1	77	G	4.251
PTK2	544	HIS	NRSN2-AS1	78	U	4.264
PTK2	599	PHE	NRSN2-AS1	76	U	4.285
PTK2	444	PRO	NRSN2-AS1	35	C	4.357
PTK2	595	ASN	NRSN2-AS1	75	A	4.399
PTK2	473	LEU	NRSN2-AS1	77	G	4.408
PTK2	535	ALA	NRSN2-AS1	82	U	4.446
PTK2	415	TYR	NRSN2-AS1	36	A	4.509
PTK2	599	PHE	NRSN2-AS1	78	U	4.516
PTK2	596	PHE	NRSN2-AS1	72	U	4.559
PTK2	479	ASP	NRSN2-AS1	36	A	4.563
PTK2	415	TYR	NRSN2-AS1	77	G	4.574
PTK2	597	ARG	NRSN2-AS1	73	U	4.576
PTK2	470	GLN	NRSN2-AS1	77	G	4.616

Receptor name	Receptor position	Receptor element	Ligand name	Ligand position	Ligand element	Distance (Å)
PTK2	466	GLU	NRSN2-AS1	77	G	4.695
PTK2	597	ARG	NRSN2-AS1	75	A	4.783
PTK2	600	THR	NRSN2-AS1	73	U	4.784
PTK2	661	TYR	NRSN2-AS1	71	U	4.792
PTK2	592	GLU	NRSN2-AS1	76	U	4.837
PTK2	487	ILE	NRSN2-AS1	36	A	4.846
PTK2	538	GLU	NRSN2-AS1	82	U	4.947
PTK2	594	ILE	NRSN2-AS1	38	A	4.973

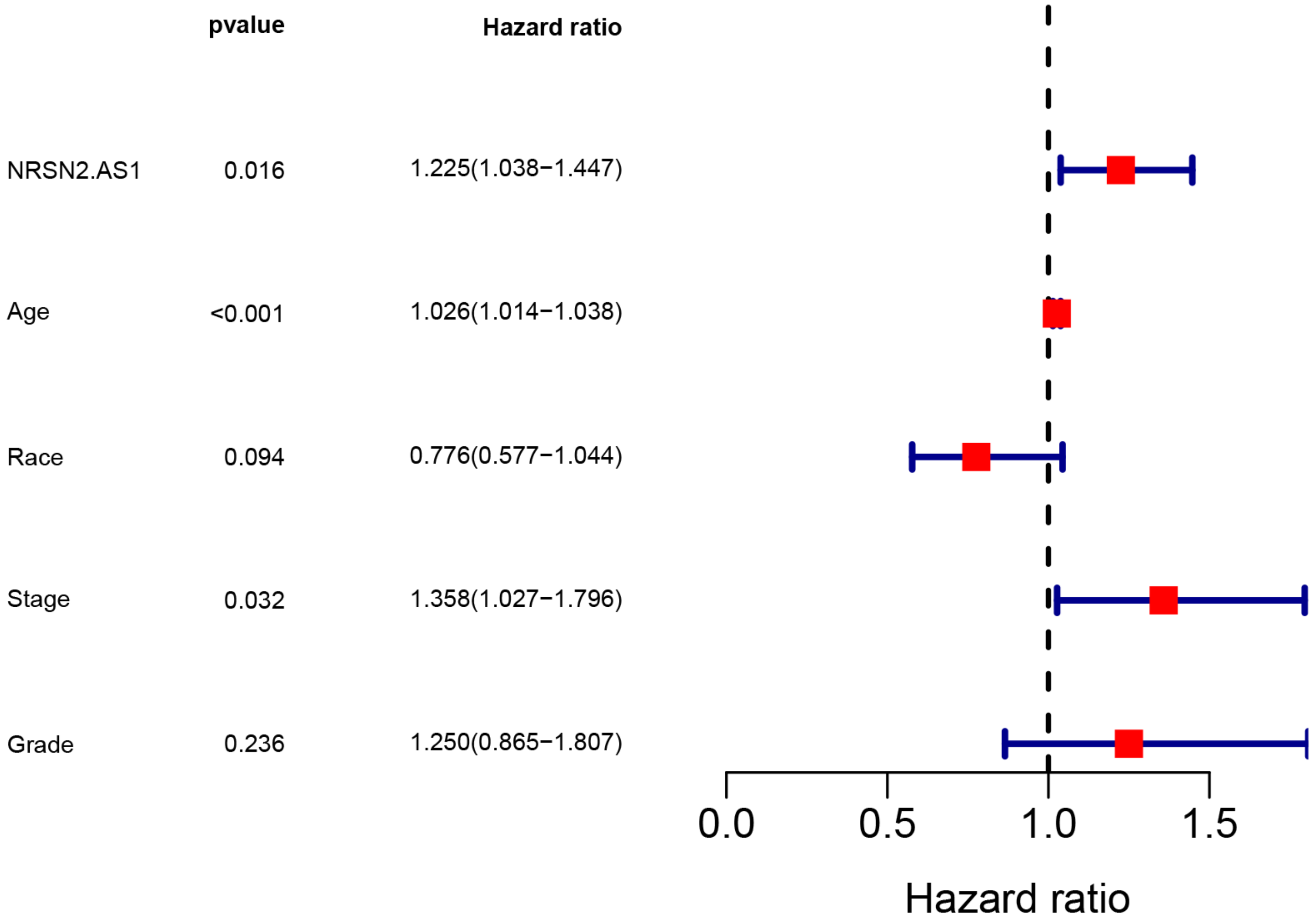

Figure S1: The results of multivariate Cox regression analysis.

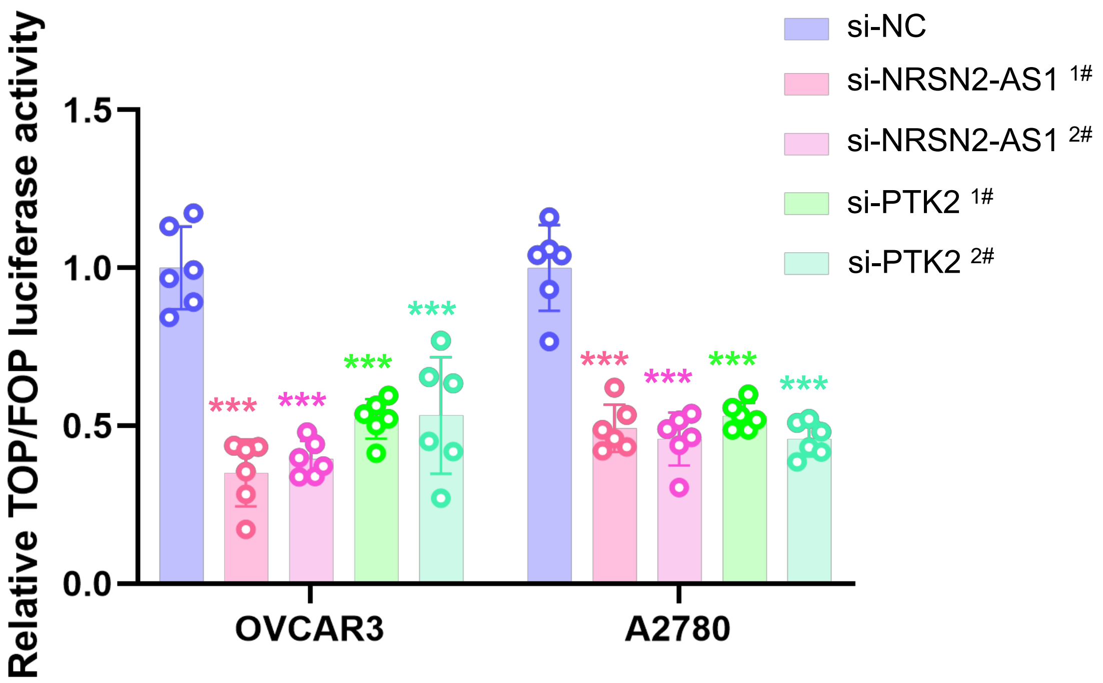

Figure S2: NRSN2-AS1/PTK2 regulates β-catenin signaling pathway in ovarian cancer. TOP/FOP flash luciferase assays performed in OC cells, n = 3. *P < 0.05, **P < 0.01, ***P < 0.001.

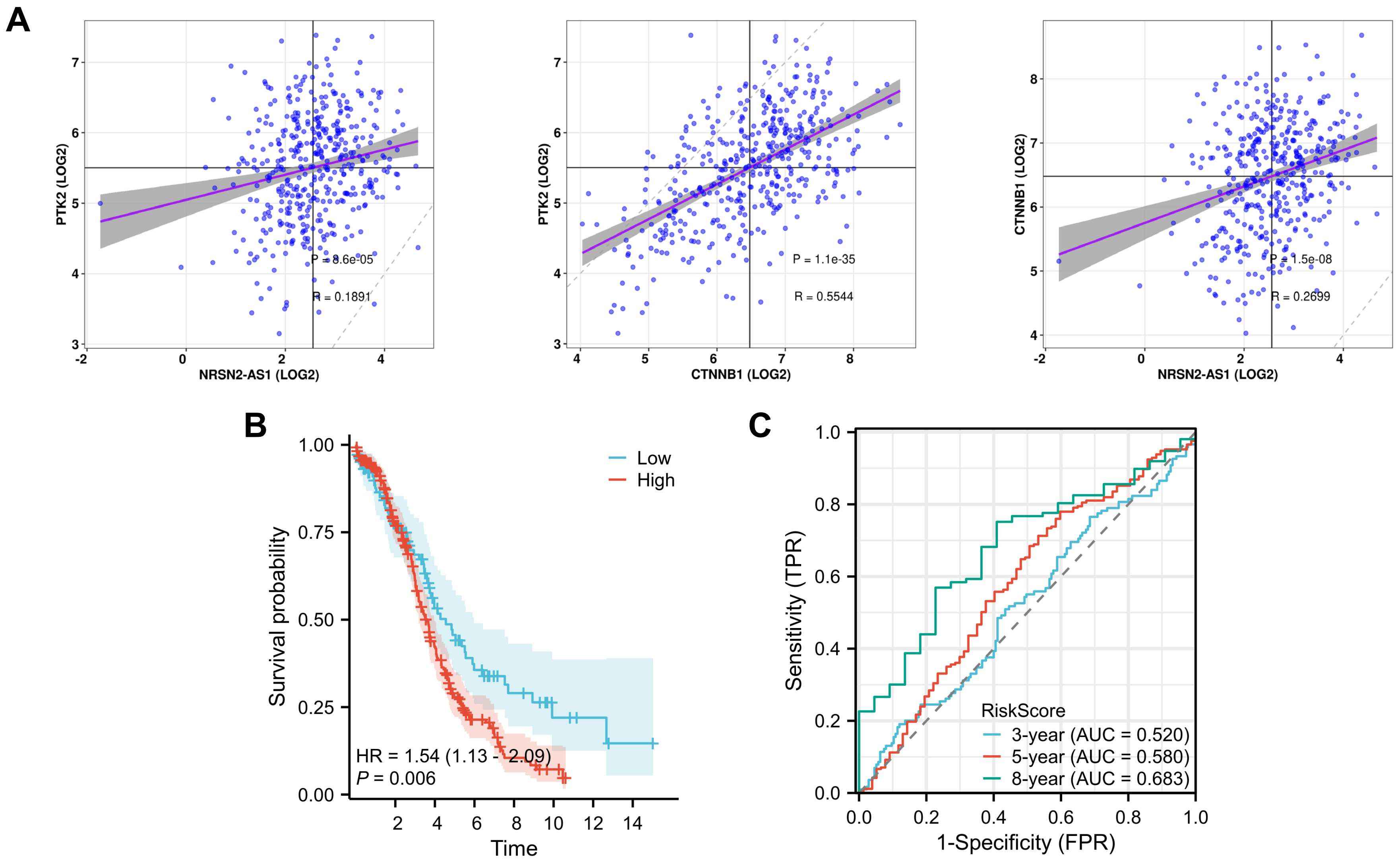

Figure S3: NRSN2-AS1/PTK2/β-catenin axis was associated with poor prognosis of OC patients. (A) The correlation between NRSN2-AS1 and PTK2, PTK2 and β-catenin, NRSN2-AS1 and β-catenin based on TCGA. **(B)** Kaplan–Meier survival curve of the integrated NRSN2-AS1/PTK2/β-catenin gene signature. **(C)** Time-dependent receiver operating characteristic (ROC) curve of the the integrated NRSN2-AS1/PTK2/β-catenin gene signature at 3, 5 and 8 years based on TCGA.

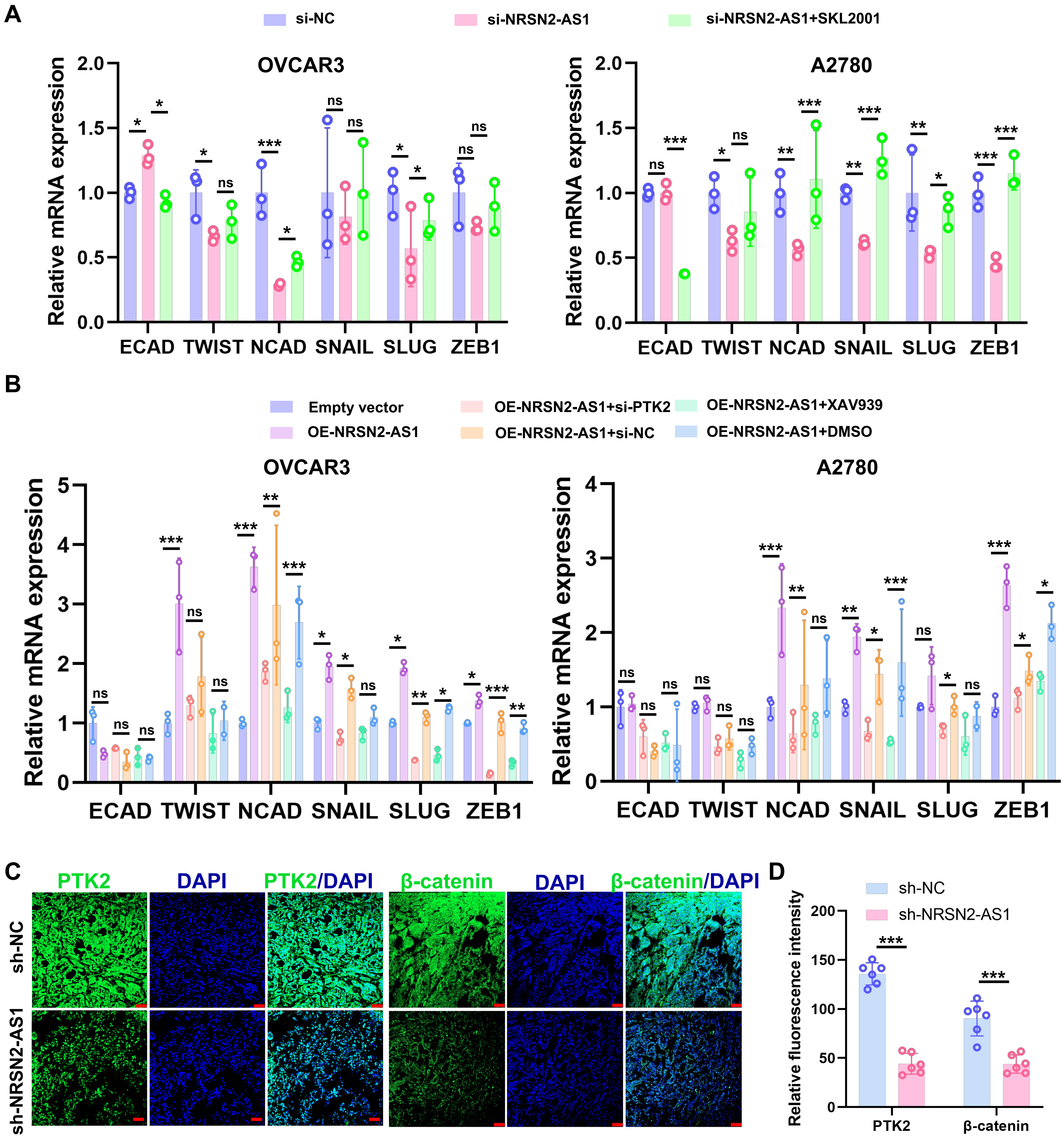
A
si-NC
si-NRSN2-AS1
si-NRSN2-AS1+SKL2001
OVCAR3
A2780
Relative mRNA expression
ECAD TWIST NCAD SNAIL SLUG ZEB1
B
Empty vector
OE-NRSN2-AS1
OE-NRSN2-AS1+si-PTK2
OE-NRSN2-AS1+si-NC
OE-NRSN2-AS1+XAV939
OE-NRSN2-AS1+DMSO
OVCAR3
A2780
Relative mRNA expression
ECAD TWIST NCAD SNAIL SLUG ZEB1
C
PTK2
DAPI
PTK2/DAPI
β-catenin
DAPI
β-catenin/DAPI
sh-NC
sh-NRSN2-AS1
D
sh-NC
sh-NRSN2-AS1
Relative fluorescence intensity
PTK2
β-catenin

Figure S4: The effect of NRSN2-AS1/PTK2/β-catenin axis on ovarian cancer cells. (A) The relative expression of EMT markers in OC cells treated with si-NC, si-NRSN2-AS1 or si-NRSN2-AS1+ SKL2001, n = 3. **(B)** The RT-qPCR analyses of EMT markers in OC cells treated with Empty vector, OE-NRSN2-AS1, OE-NRSN2-AS1+ si-PTK2, OE-NRSN2-AS1+ si-NC, OE-NRSN2-AS1+XAV939 or OE-NRSN2-AS1+ DMSO, n = 3 for each group. **(C)** Immunofluorescence assays showed the expression of PTK2/β-catenin (green) in tumors derived from OC cells transfected with sh-NC/sh-NRSN2-AS1, with nuclei stained by DAPI (blue). Scale bar: 50 μm. (D) Quantification of fluorescence intensity of (C), n = 6. *P < 0.05, **P < 0.01, ***P < 0.001.
