# Supplementary figures and images for: Long non-coding RNA NRSN2-AS1 promotes ovarian cancer progression through targeting PTK2/β-catenin pathway
Source: Cell Death Dis. 2023 Oct 24;14(10):696. doi: 10.1038/s41419-023-06214-z (PMC10598275; doi:10.1038/s41419-023-06214-z)

Figure 2H

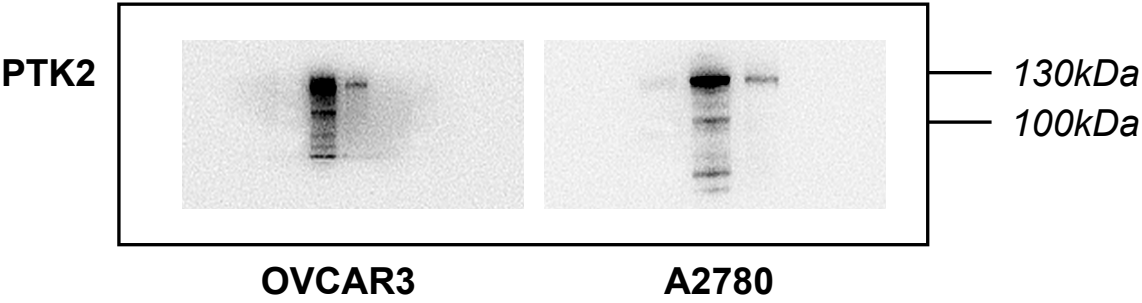

Figure 3H

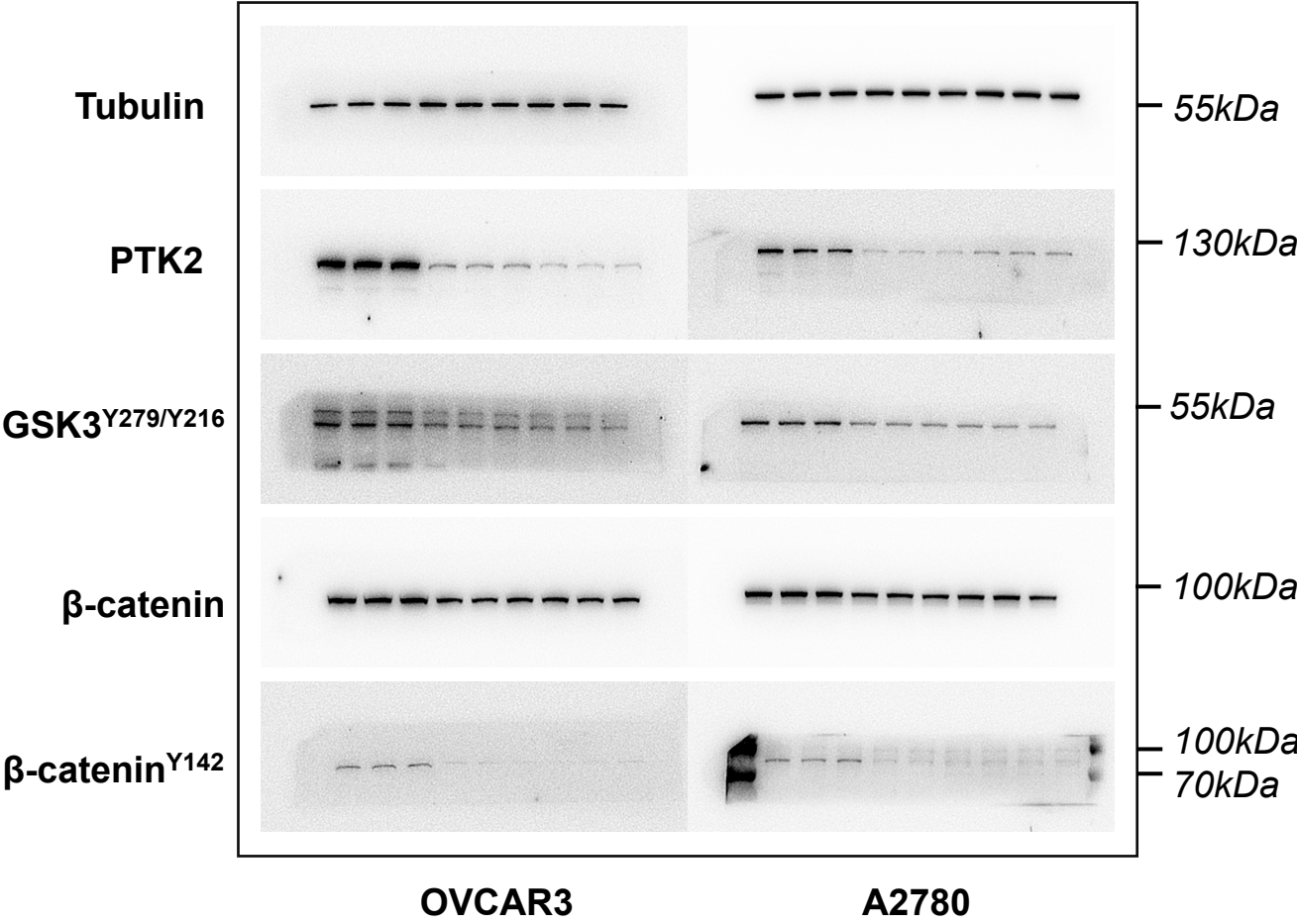

Figure 4A

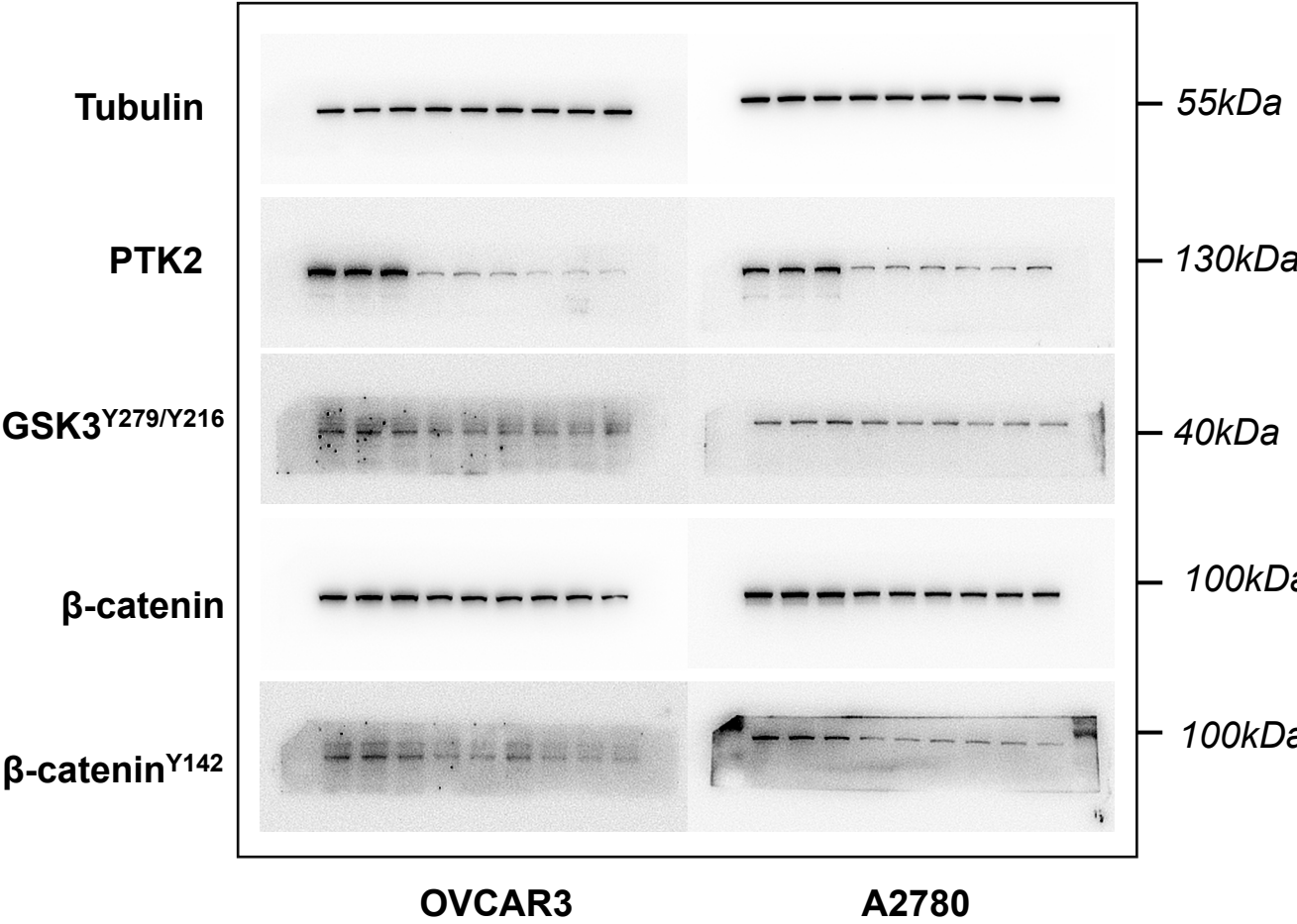

Figure 5A

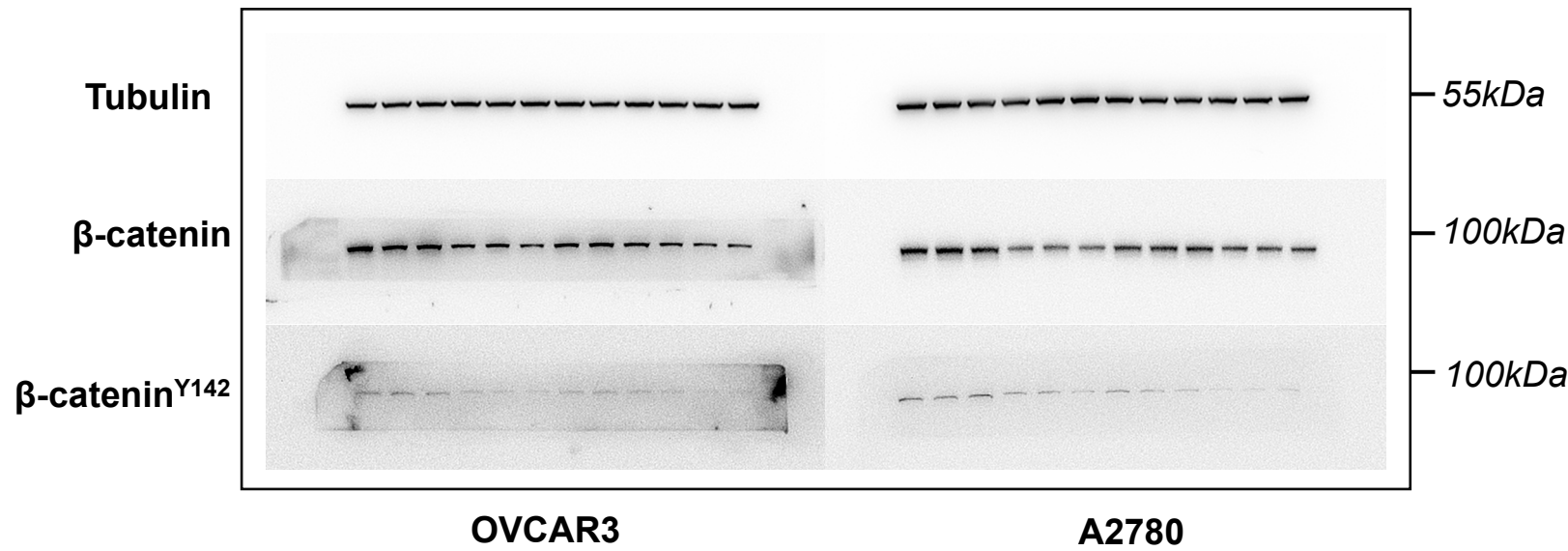

Figure 6A

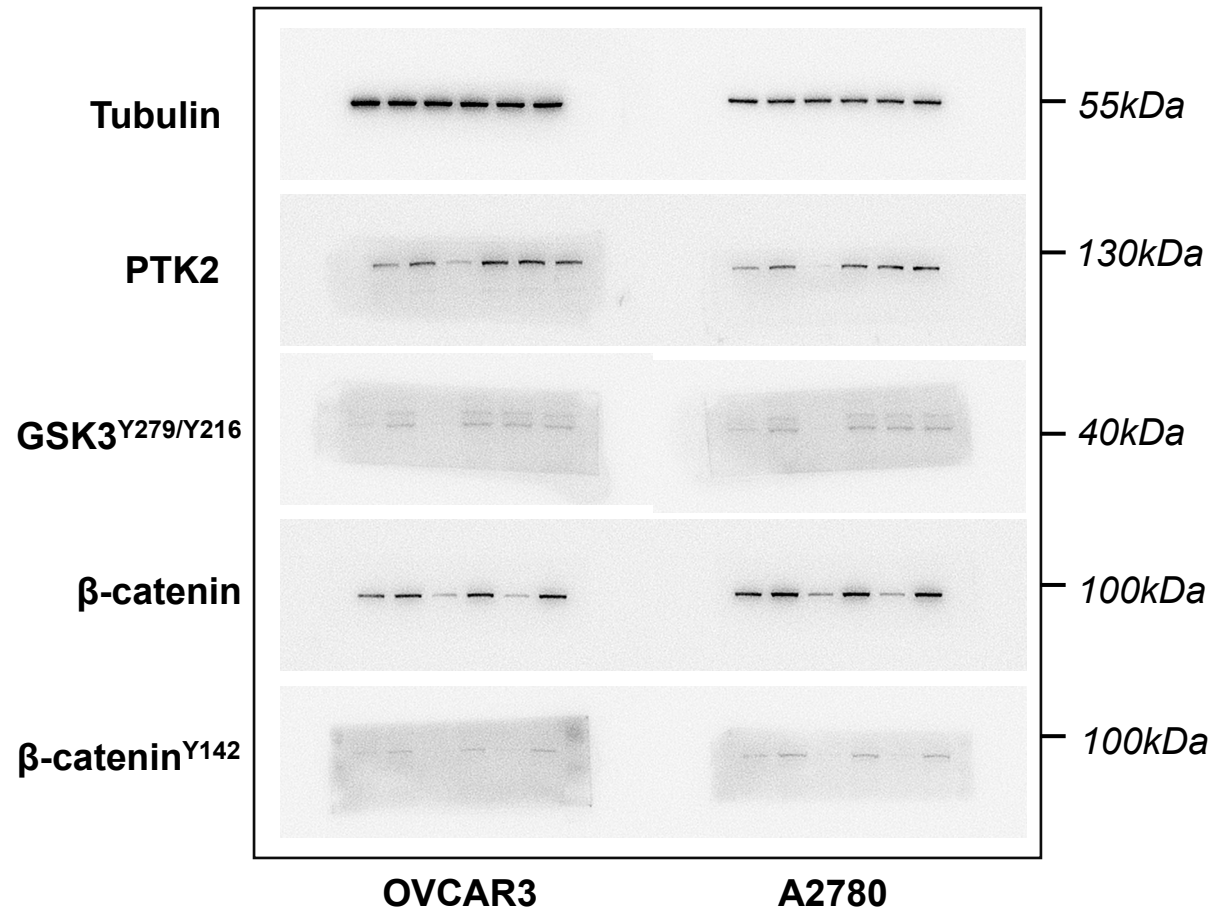

Figure 7A

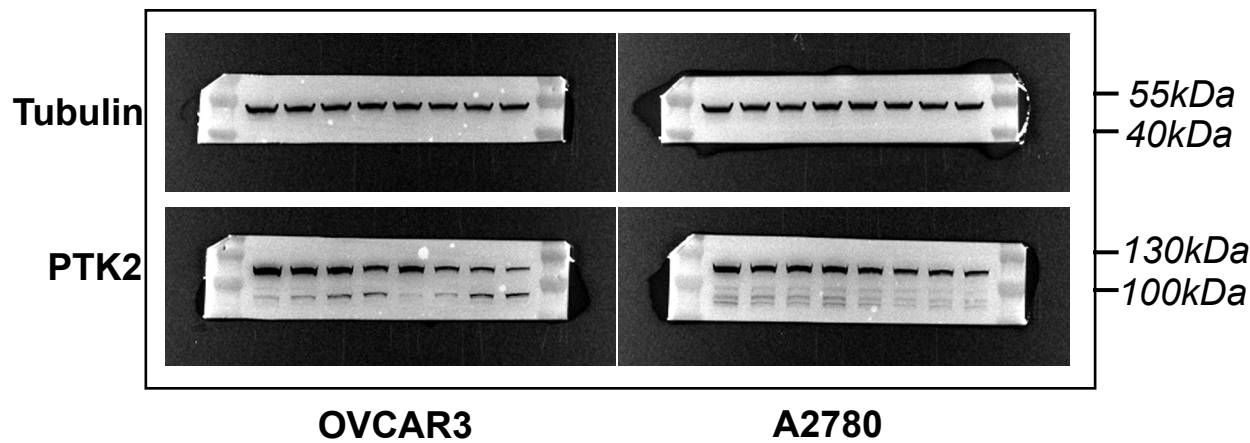

Figure 7N

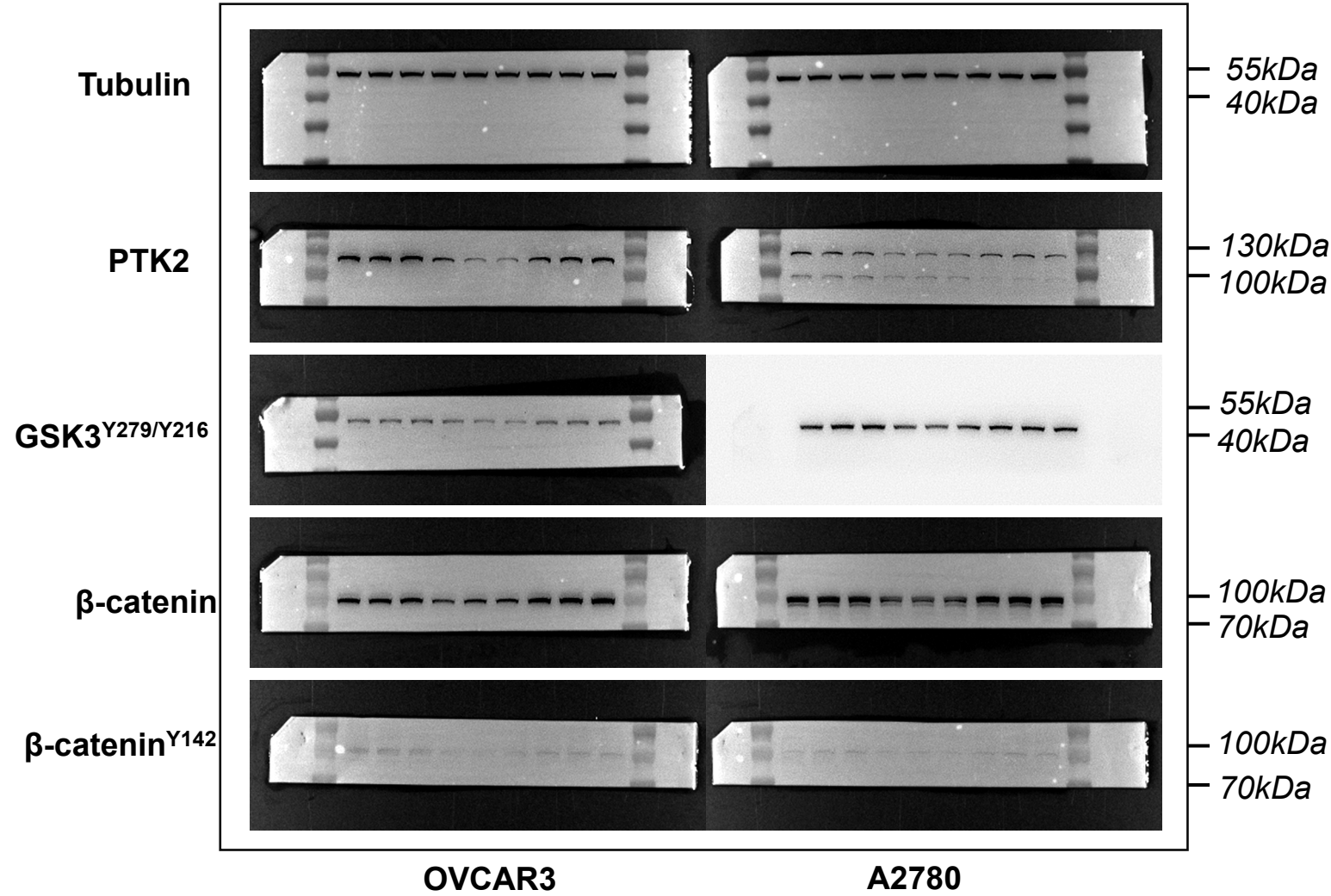

Figure 7P

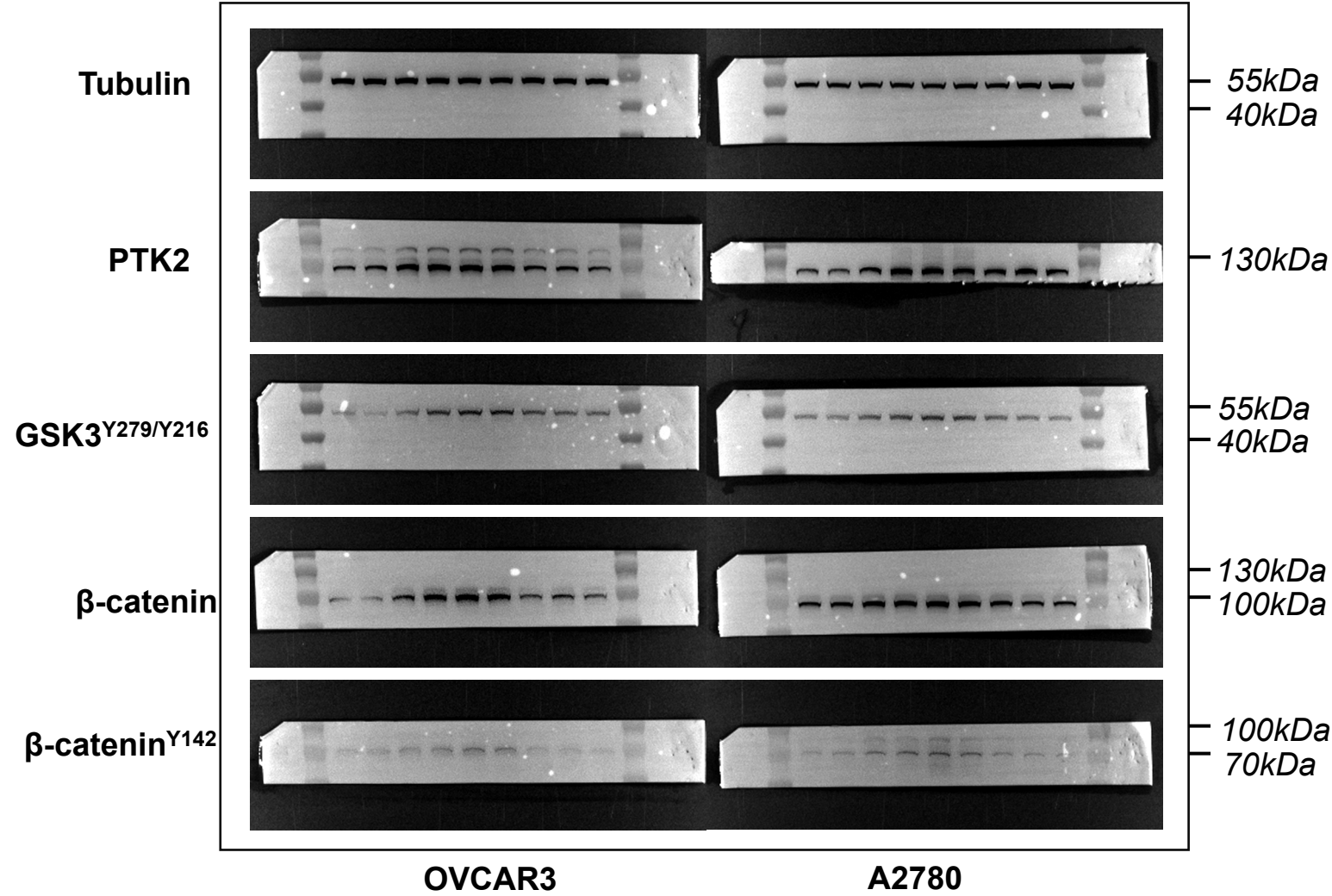

Supplement: Supplementary file 2 — Origial Data File [file 41419_2023_6214_MOESM2_ESM.pdf]
